# Multiscale simulation of salt crystallization-induced damage in porous materials

**DOI:** 10.1617/s11527-025-02709-7

**Published:** 2025-07-17

**Authors:** N. Lo Presti, A. M. D’Altri, L. Patruno, G. Castellazzi, H. Derluyn, S. de Miranda

**Affiliations:** 1 Department of Civil, Chemical, Environmental, and Materials Engineering (DICAM), University of Bologna, Viale del Risorgimento 2, 40136 Bologna, Italy; 2 Université de Pau et des Pays de l’Adour, E2S UPPA, CNRS, LFCR, Pau, France

**Keywords:** Pore pressure, Micromechanics, Pore structure, Finite element analysis, Damage modelling

## Abstract

In this paper, a multiscale modelling strategy to simulate salt crystallization-induced damage in porous materials is proposed. Salt crystallization pressure exerted on pore walls is explicitly modelled on a nonlinear representative volume element (RVE) at the microscale of the porous medium. A macroscopic damage measurement of the whole RVE can be then extracted for any combination of crystallization pressure and pore filling time histories. The efficient coupling of moisture transport and salt crystallization with micromechanical damage is achieved by adopting a state-of-the-art multiphase model for the transport/crystallization part and by originally formulating an efficient phenomenological damage model, trained on a dataset generated through micromechanics-based simulations on RVEs. The effectiveness of this numerical strategy is shown via the comparison with an experimental campaign on salt-aged traditional Dutch tiles. The proposed numerical strategy appeared able to track the evolution of macroscopic damage in real-time along with salt transport and crystallization within the porous medium. The potential for using the proposed framework with extended datasets and simulation-driven machine learning is also highlighted.

## Introduction

Salt crystallization-induced damage is one of the main causes of environmental ageing in stones and other porous building materials as recognized by the scientific community [1–10]. According to climate change predictions, ageing effects of salt weathering in porous media are even expected to worsen in the future [11, 12]. Accordingly, comprehensive knowledge about the chemo-mechanical processes of salt crystallization-induced damage in porous materials would be desirable. However, such phenomena are inherently multi-physical and characterized by multiple scales, ranging from the pore size to the structural one, so that an effective simulation strategy requires to bridge the two in a simple, yet representative way. For such reason, this research issue is still a hot topic in the scientific community [10, 13–15]. The resistance of porous building materials against salt crystallization-induced ageing is typically tested experimentally through accelerated test protocols [16–20]. Despite these advances, it appears still challenging to reproduce long-term actual weathering conditions in laboratory.

To fill this gap, numerical tools to simulate long-term weathering conditions and salt crystallization-induced damage in porous media have been developed in the last years [21–23]. An example of coupled modelling of heat, moisture and salt transport in porous building materials has been introduced in [24], later extended in [25, 26] to account for salt phases changes and salt crystallization, respectively. In addition, a multiphysics model for spalling prediction in brick masonry due to in-pore salt crystallization has been developed in [27]. Another example of a computational model coupling heat, water and salt ion transport, salt crystallization, deformation and damage in porous materials has been introduced in [28]. A multiphase model for the analysis of moisture and salts transport and the prediction of stress induced by salt crystallization in masonry walls has been introduced in [29] and utilized for various applications in [30–32] as well as extended to consider also the hydration of salts in [33]. Additionally, a coupled chemo-hydro-mechanics approach based on phase-field modeling for cracking and damage induced by salt crystallization in pores has been developed in [34], based on macroscopic mechanical properties.

All these approaches foresee damage based on the macroscopic tensile strength of the material (i.e., a mechanical property at the macroscale). Accordingly, they appear to simplify considerably the mechanical problem (for example, no confinement of the surrounding material is contemplated) which actually has a micromechanical nature, being the crystallization pressure exerted at the pore structure. For this reason, micromechanical modelling approaches which can account for the microstructure of the porous material might represent a valid and accurate solution to explicitly track salt crystallization-induced damage at the micro-scale. A first step in this direction has been undertaken in [35], where a multi-scale approach based on the real 3D micro geometry of the porous material coming from X-ray micro computed tomography images has been developed to estimate the partial Biot’s coefficient as function of the degree of saturation of salt crystals. In [35], the 3D representative volume element (RVE) has been assumed as linear elastic, so allowing for stress prediction without damage estimations.

In this paper, a multiscale modelling strategy to simulate salt crystallization-induced damage in porous materials is proposed. Salt crystallization pressure exerted on pore walls is explicitly modelled on a nonlinear RVE at the microscale of the porous medium, accounting for the confinement provided by the surrounding material. A macroscopic damage measurement of the whole RVE is extracted for any combination of crystallization pressure and pore filling time histories. The efficient coupling of salt transport and crystallization with micromechanical damage is achieved by adopting the multiphase model proposed in [29] for the transport/crystallization part and by originally formulating a phenomenological damage model, trained on a dataset generated through micromechanics-based simulations on the nonlinear RVE. The effectiveness of this numerical strategy is tested via the comparison with an experimental campaign on salt-aged traditional Dutch tiles [36], carried out within the framework of the H2020-funded JPI Cultural Heritage CRYSTINART project [37].

The paper is structured as follows. Section 2 deals with the methodology developed in this paper. In particular, Sect. 2.1 briefly outlines the key aspects of the proposed numerical approach to simulate water and salt transport, salt crystallization and macroscopic damage in porous media. Section 2.2 recalls the main features of the multiphase model in [29] to simulate water, salt transport, and salt crystallization. Section 2.3 deals with the micromechanics-based damage model, where the micromechanical modelling assumptions, the procedure to extract macroscopic damage, and the development of a phenomenological damage model are presented and described. Moreover, Sect. 3 presents and discusses numerical results and their comparison with experimental outcomes, using as reference [36]. Finally, Sect. 4 collects the conclusions of this research.

## Methodology

### Numerical strategy

**Fig. 1:**
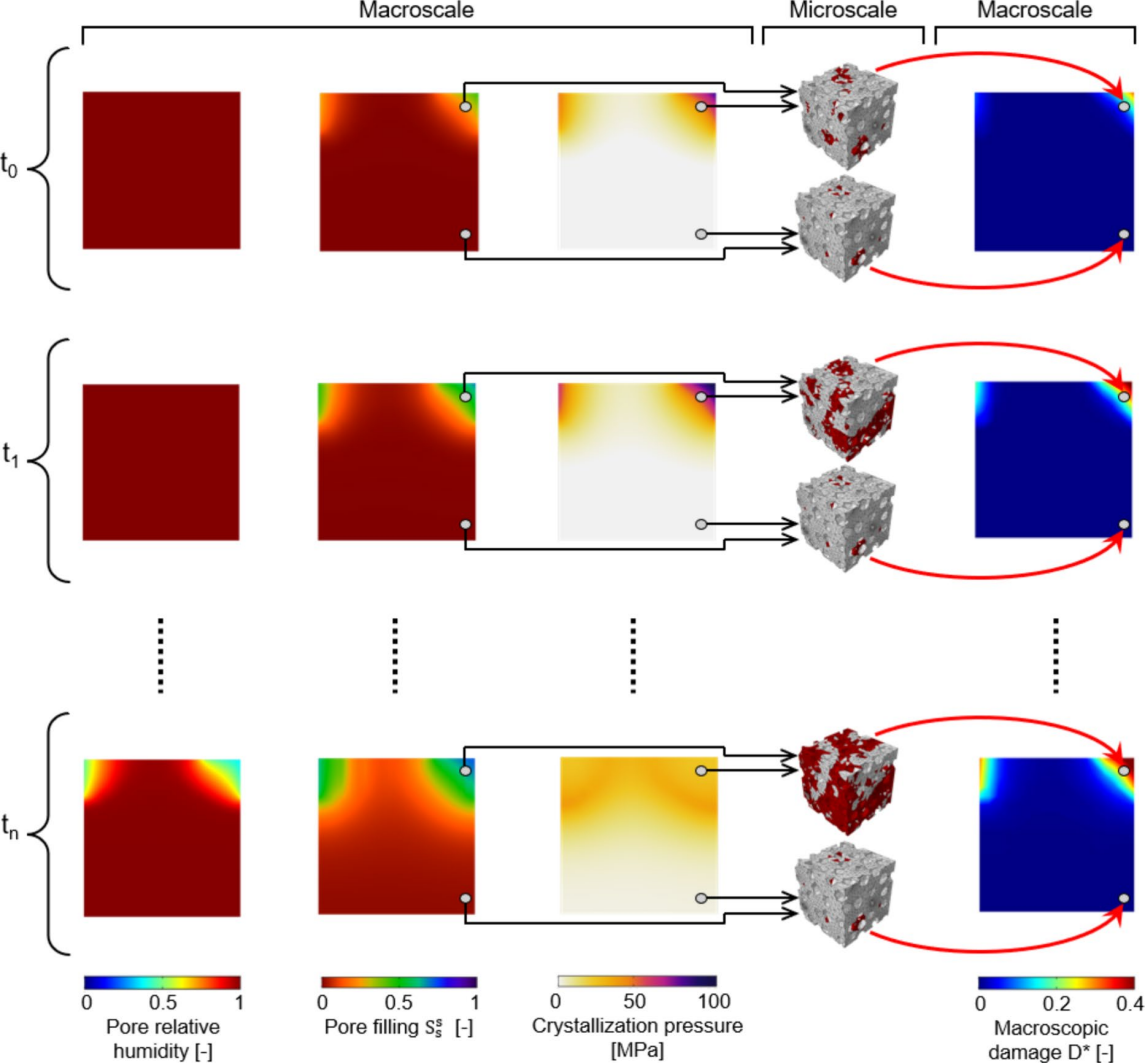
Coupling moisture transport and salt crystallization with micromechanical damage. Red parts in the RVEs highlight damage at the microscale. Macroscopic damage contour plots collect point-by-point micromechanical damage information. Details of the numerical examples are given in Sect. 3

The main idea of the multiscale modelling strategy here proposed to simulate salt crystallization-induced damage in porous materials consists in the coupling of the multiphase model introduced in [29] for the analysis of moisture transport and salt crystallization with a micromechanical damage model with the explicit representation of pore walls loaded through crystallization pressure (Fig. 1). In particular, the multiphase model simulates moisture transport and salt crystallization at the macroscale. Accordingly, pore filling and crystallization pressure time histories are post-processed from each integration point of the domain (macroscale). Thus, pore filling and crystallization pressure time histories of each integration point are used as input in the micromechanical damage model.

The micromechanical damage model consists of a 3D RVE with pores explicitly modelled as voids within a conforming solid finite element (FE) mesh, where the FEs obey to an isotropic plastic-damage constitutive law [38] to account for damage at the microscale. Pore walls of the RVE are loaded according to the pore filling and crystallization pressure time histories extracted from the multiphase model. The microscale nature of the RVE allows to account for the pore filling in a direct way, i.e., only a part of randomly distributed pores (equal to the pore filling) is loaded. The (eventually damaged) RVE is then de-loaded from crystallization pressure and a macroscopic damage measurement of the whole RVE is extracted mimicking a compression test, i.e., the relative variation of stiffness from an initially undamaged RVE is computed. Technically, this operation should be performed for any integration point of the macroscopic domain. However, the macroscopic domain of a porous building material might be characterized by thousands of integration points, and nonlinear static analyses on the RVEs might require a simulation time up to a few hours on a standard computer (see, e.g., Appendix 1). Accordingly, thousands of RVE-based nonlinear micromechanical analyses might result computationally extremely expensive, leading the workflow to be impractical in most cases.

To solve this issue, an ad hoc efficient phenomenological damage model is developed, specifically trained (and validated) by means of a dataset generated through RVE-based nonlinear micromechanical analyses. Therefore, the phenomenological damage model receives as input the pore filling and crystallization pressure time histories of an integration point and (in real-time) gives as output the corresponding estimated macroscopic damage. Accordingly, the micromechanics-based macroscopic damage time history is reconstructed for each integration point of the macroscopic domain, so obtaining a macroscopic damage field for any time instant (Fig. 1). Thereby, the output of the multiphase model (pore relative humidity, pore filling, and crystallization pressure contour plots) is integrated in real-time with mechanical damage at the macroscale.

### Modeling of moisture transport and salt crystallization

The multiphase model for the simulation of moisture transport and salt crystallization in porous building materials at the macroscale is here briefly recalled. Further details can be found in [29, 31]. In this modelling approach, the porous material is idealized as a multiphase continuous porous medium composed of the solid material matrix, gaseous and/or liquid water, and liquid and/or solid salt. With the hypotheses of isothermal conditions and a unique salt solid phase (sodium chloride in this case), the multiphase model is formulated in terms of the three independent variables (i) pore relative $h$ (ratio between the actual vapor pressure and the vapor pressure at saturation), (ii) mass fraction of the dissolved salt $\omega$, and (iii) concentration of crystallized salt $c_{s}^{s}$. According to [29, 31], the multiphase model is described through a moisture mass conservation equation, a salt mass conservation equation, as well as through an evolution equation defining the kinetics of salt precipitation/dissolution, hypothesizing an isotropic distribution of cylindrical pores and cylindrical nuclei of the same radius of the pores ($r_{p}$). Particularly, the supersaturation ratio $\omega /\omega_{{{\mathrm{sat}}}}$ is assumed to rule the evolution equation, i.e. crystallization occurs when $\omega /\omega_{{{\mathrm{sat}}}}$ overtakes a pre-defined threshold and dissolution occurs when $\omega /\omega_{{{\mathrm{sat}}}}$ is lower than one (with $\omega_{{{\mathrm{sat}}}}$ the mass of dissolved salt per unit mass of liquid phase at saturation).

Being ${\mathbf{j}}_{w}$ the water flux (${\mathbf{j}}_{w} = {\mathbf{j}}_{w}^{g} + {\mathbf{j}}_{w}^{l}$, with ${\mathbf{j}}_{w}^{g}$ the water vapor flux and ${\mathbf{j}}_{w}^{l}$ the water liquid flux), and ${\mathbf{j}}_{s}^{l}$ the flux of dissolved salt, and considering that the fluxes ${\mathbf{j}}_{w}^{l}$ and ${\mathbf{j}}_{s}^{l}$ can be expressed as ${\mathbf{j}}_{w}^{l} = \left( {1 - \omega } \right){\mathbf{j}}_{ws}^{l} - {\mathbf{j}}_{{s,{\mathrm{diff}}}}^{l}$ and ${\mathbf{j}}_{s}^{l} = \omega {\mathbf{j}}_{ws}^{l} + {\mathbf{j}}_{{s,{\mathrm{diff}}}}^{l}$, being ${\mathbf{j}}_{ws}^{l}$ the flux of the liquid phase and ${\mathbf{j}}_{{s,{\mathrm{diff}}}}^{l}$ the diffusive flux of the dissolved salt, the constitutive relationships for water vapor flux ${\mathbf{j}}_{w}^{g}$, the capillary liquid flux ${\mathbf{j}}_{ws}^{l}$ and the diffusive flux of dissolved salt ${\mathbf{j}}_{{s,{\mathrm{diff}}}}^{l}$ are defined, according to [29, 31], through the vapor permeability $K_{g}$, the liquid permeability of the salt solution $K_{l}$, and the salt diffusion coefficient $K_{s}$. Details about the settings of $K_{g}$, $K_{l}$, and $K_{s}$ can be found in [29, 31].

In this multiphase model, the salt solution saturation degree $S_{ws}^{l}$ is written as function of the relative humidity $h$ through the sorption/desorption curve $S_{ws}^{l} \left( h \right)$, which is typically derived experimentally. The model is finally completed by the boundary conditions, which can have four different forms:

1 $$h = \overline{h} , \quad \quad \omega = \overline{\omega } , \quad \quad{\mathbf{j}}_{w} \cdot {\mathbf{n}} = \gamma_{w} \left( {A_{w} h - h_{env} } \right) ,\quad \quad {\mathbf{j}}_{s}^{l} \cdot {\mathbf{n}} = 0$$

where ${\mathbf{n}}$ is the outward unit normal to the boundary, $\overline{h}$ and $\overline{\omega }$ the prescribed humidity and salt concentration, respectively, $h_{{{\mathrm{env}}}}$ the prescribed environmental humidity,$A_{w}$ the water activity, and $\gamma_{w}$ the convective humidity coefficient. A standard iterative strategy based on the Newton–Raphson method is adopted to solve the non-linear system of differential equations. The time discretization is performed through the backward finite difference method.

In post-processing, the pore volume filled by precipitated salt $S_{s}^{s}$, which corresponds to the pore filling with respect to the capillary porosity, is straightforwardly given by:

2 $$S_{s}^{s} = \frac{{c_{s}^{s} }}{{\phi_{0} \rho_{s}^{s} }}$$

being $\rho_{s}^{s}$ the solid salt density, and ϕ_{0} the capillary active porosity of the material. Also, an estimation of the crystallization pressure p can be extracted using state-of-the-art expressions [39–41], e.g.:

3 $$P = \frac{{\nu {{RT}}}}{{V_{s} }}\left( {\ln \frac{\omega }{{\omega_{{{\mathrm{sat}}}} }} + \ln \frac{\gamma }{{\gamma_{{{\mathrm{sat}}}} }}} \right)$$

where R is the ideal gas constant (8.31 × 10^−3^ kJ/mol/K), V_(s) the molar volume of solid salt, $\nu$ the total number of ions released upon complete dissociation of the salt, and $\frac{\gamma }{{\gamma_{{{\mathrm{sat}}}} }}$ the normalized mean activity coefficient which can be evaluated, e.g., through an ion interaction approach as suggested in [39]. It is worth to note that any suitable relationship to estimate the crystallization pressure can be used in place of Eq. (3), as the present numerical strategy is general. The multiphase problem is solved by means of the FE method, the domain being subdivided into quadrangular FEs with quadratic shape functions, and the time integration being performed by means of the backward differentiation formula implicit method. The multiphase model is implemented in COMSOL Multiphysics [42].

### Micromechanics-based damage model

#### Micromechanical modeling assumptions

3D nonlinear RVEs are developed to relate crystallization pressure $P$ and pore filling $S_{s}^{s}$ to damage in a material point, using a micromechanical framework. The idea is then to explicitly simulate salt crystallization pressure exerted on pore walls (in a number of pores proportional to $S_{s}^{s}$) and to extract an overall information about damage on the RVE. For this reason, a nonlinear damaging RVE is supposed.

The geometry of the RVEs is meant to be statistically representative of the porous material [43–47]. Accordingly, mean pore radius $r_{p}$ and capillary active porosity $\phi_{0}$ are used as target properties to generate the geometry of the RVE composed of randomly located spherical pores. As rule of thumb, cubic RVEs with edge sizes 10 times larger than $r_{p}$ are considered. Such assumption has been found consistent in preliminary studies about RVE size, see an example in Appendix 1. In this study, non-superimposing spherical pores with identical radius are considered for simplicity. This assumption has been also found reasonable in preliminary tests on RVE pore geometries, see an example with different pore size distributions in Appendix 1.

The material response of the porous material solid matrix is supposed to follow an isotropic plastic-damage constitutive model [38]. Quasi-brittle behaviours are assumed in both compressive $\sigma_{c} = \left( {1 - d_{c} } \right)E\left( {\varepsilon_{c} - \varepsilon_{c}^{p} } \right)$ and tensile $\sigma_{t} = \left( {1 - d_{t} } \right)E\left( {\varepsilon_{t} - \varepsilon_{t}^{p} } \right)$ uniaxial regimes, by recurring to two independent damage variables for compression ($0 \le d_{c} < 1$) and tension ($0 \le d_{t} < 1$), being $E$ the initial Young’s modulus of the pore structure, $\sigma_{c}$ and $\sigma_{t}$ the uniaxial compressive and tensile stresses, $\varepsilon_{c}$ and $\varepsilon_{t}$ the uniaxial compressive and tensile strains, and $\varepsilon_{c}^{p}$ and $\varepsilon_{t}^{p}$ the respective plastic strains. A nonassociative flow rule is adopted to govern dilatancy (with an angle of 10°) and to set the plastic strain rate, while a multiple-hardening Drucker-Prager type surface is used as yielding surface. Beyond general parameters for quasi-brittle materials to set the strength domain, the constitutive model for the pore structure is primarily characterized by compressive and tensile strengths ($f_{c}$ and $f_{t}$, respectively), as well as the evolution of the uniaxial post-peak responses which is assumed to reach linearly the residual strengths (i.e., a tenth of $f_{c}$ and $f_{t}$, respectively) together with damage variables which reach 0.9 (to guarantee numerical convergence) at inelastic strains equal to 0.003 and 0.001, respectively. In this study, red fired clay bricks and clay bodies of Dutch tiles are considered. The adopted material properties are highlighted in Table 1, where the target compressive and tensile strengths ($\overline{{f_{c} }}$ and $\overline{{f_{t} }}$, respectively) and target Young’s modulus ($\overline{E}$) of equivalent homogeneous materials have been assumed from the literature (for red clay bricks, with $r_{p} = 0.7 {\upmu m}$ and $\phi_{0} = 26.0\%$) and from experiments (for clay bodies of Dutch tiles [36], with $r_{p} = 0.35$ μm and $\phi_{0} = 26.6\%$). The ones used for the pore structure have been set in order to obtain the same response of the target properties on a homogeneous material.

**Table 1:** Mechanical properties of the materials considered

Material	$\overline{E}$(GPa)	$E$(GPa)	$\overline{{f_{c} }}$(MPa)	$f_{c}$(MPa)	$\overline{{f_{t} }}$(MPa)	$f_{t}$(MPa)
Red fired clay brick	7.2	10.5	20.3	58.0	3.1	6.8
Clay body of Dutch tiles	16.8	25.6	21.0	41.0	3.0	6.0

The 3D solid geometry of the pore structure is discretized by means of 4-node tetrahedral finite elements with an average element size equal to the mean pore radius $r_{p}$ (e.g., 0.7 μm). With this element size and the aforementioned inelastic strains related to a fully damaged material, the fracture energy of the pore structure tends to zero, consistently with the observations in [48]. Accordingly, the post-peak response of the pore structure appears substantially brittle, as assumed e.g. in [49, 50]. In Appendix 1, examples investigating the effect of different mesh sizes are shown, confirming the effectiveness of the proposed approach.

**Fig. 2:**
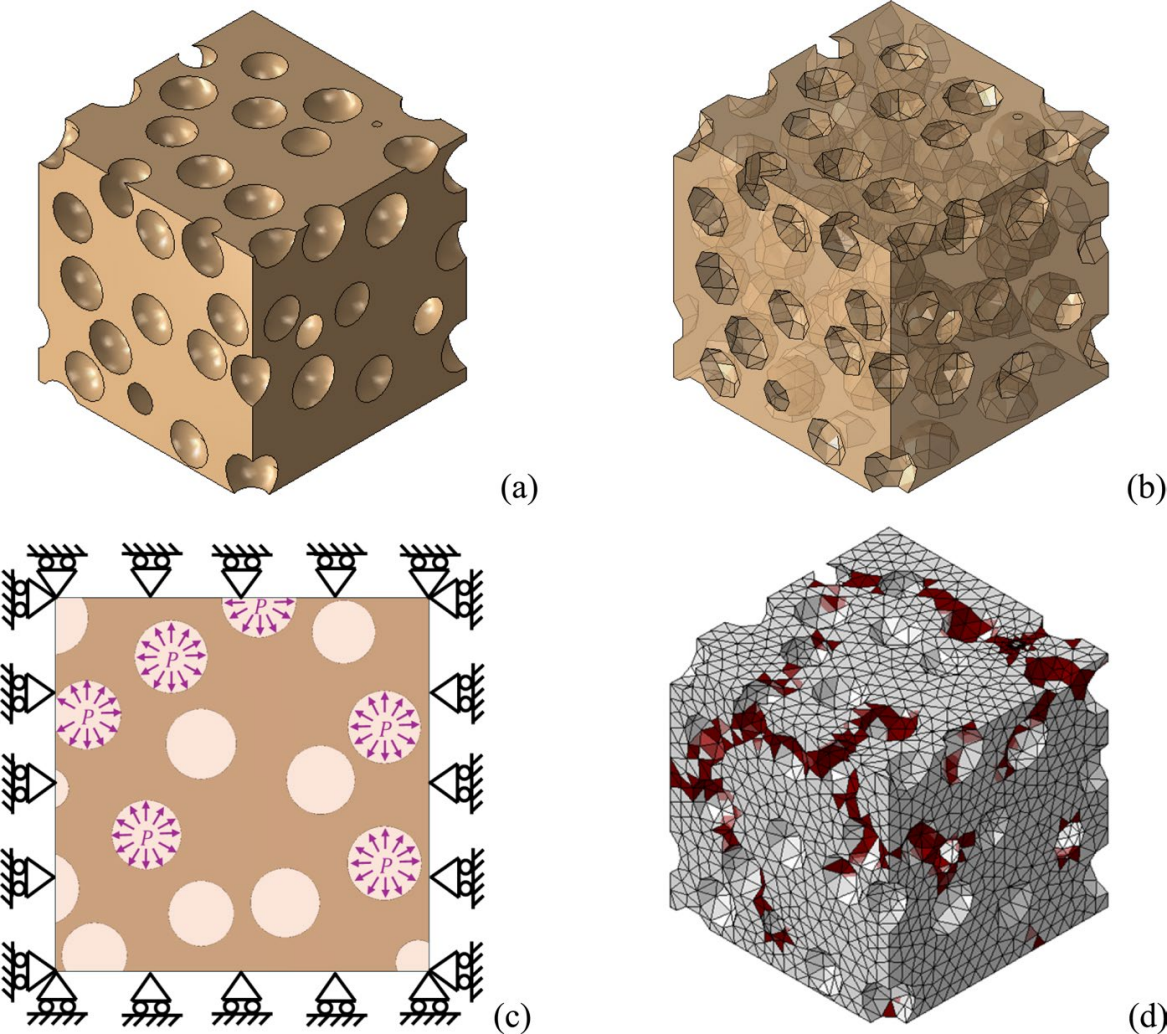
Micromechanics-based damage model. **a** Example of a $7$ μm-RVE solid geometry. **b** Discretized geometry with transparency to highlight the inner pores. **c** Schematic 2D representation of loads and boundary conditions of the RVE. **d** Example of a resulting damaged RVE, with red elements highlighting damage (with $0.3 \le d_{c} \le 0.9$), showing an example of FE mesh

Salt crystallization pressure is explicitly modelled in the RVE through a normal pressure exerted on the pore walls (Fig. 2). Such pressure $P$ can be monotonically increased as well as varied cyclically along with the pseudo-time of the simulation, in a nonlinear static analysis framework. The percentage of pores loaded with pressure in the RVE can be directly related to the pore filling $S_{s}^{s}$. As way of example, with $S_{s}^{s} = 0.4$ only 40% of pores randomly distributed will be loaded with pressure. The distribution of loaded pores has a small influence on results, as shown in Appendix 1. It should be highlighted that the law of partial pressures [35, 51] is not adopted here, as the micromechanical model allows for a direct definition of the number of pores to be loaded.

Accordingly, the RVE can be loaded with any pseudo-time history of pressure $P$ and pore filling $S_{s}^{s}$. In order to account for the confinement exerted by the material surrounding the RVE, normal displacements are constrained at the external surfaces of the RVE (Fig. 2). This hypothesis, although simplified, is expected to be representative of the material condition [35], given that a similar crystallization pressure might be hypothetically exerted into the material surrounding the RVE, with the only exception concerning the material at the very external boundary of the specimen.

#### Macroscopic damage

**Fig. 3:**
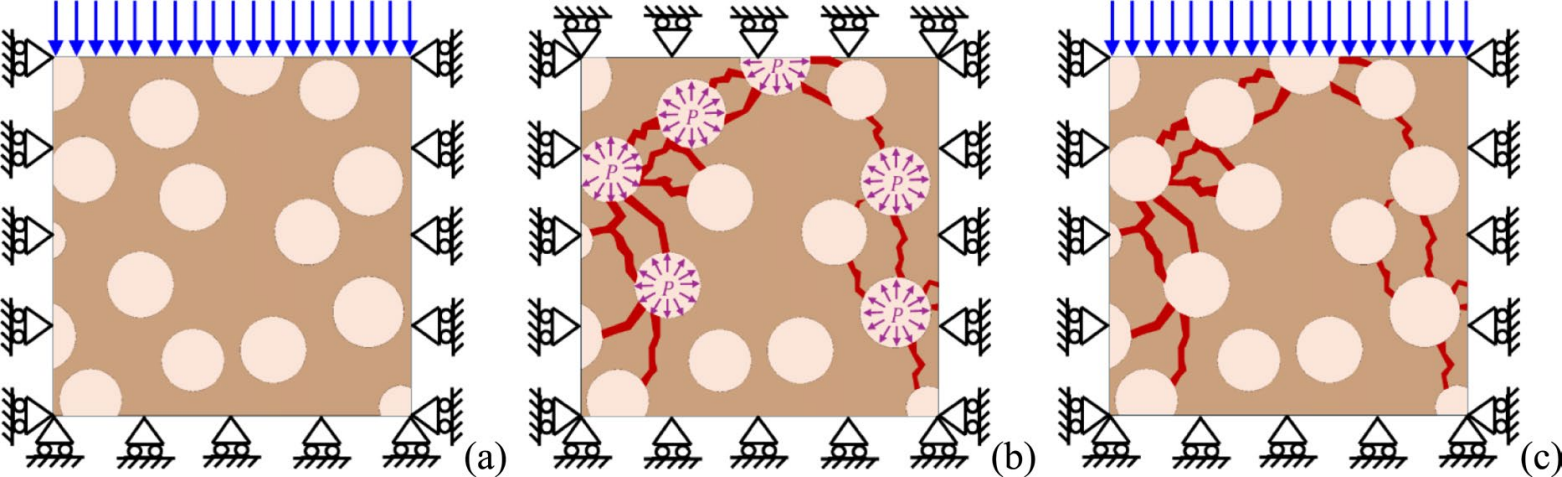
Macroscopic damage. **a** 2D schematization of a compression test on the intact RVE. **b** Application of pore pressure and corresponding damage on the RVE. **c** 2D schematization of a compression test on the damaged RVE

The RVE is subjected to a certain pseudo-time history of $P$ and $S_{s}^{s}$, which can possibly damage the pore structure. In order to extract a macroscopic damage information on the RVE, a simple compression test-based procedure is adopted, as shown in Fig. 3. Firstly, a compression test is conducted on the intact RVE, and the initial undamaged stiffness ($K^{{{\mathrm{UND}}}}$) is measured as sketched in Fig. 3a. Then, the pore pressure is applied to the pore structure of the RVE according to the selected pseudo-time history of $P$ and $S_{s}^{s}$, which might lead to damage into the RVE (Fig. 3b). The pore pressure is eventually dismissed and, finally, another compression test is conducted on the damaged RVE (Fig. 3c). Accordingly, the damaged stiffness $\left( {K^{D} } \right)$ is measured and compared with the undamaged stiffness. The percentage reduction of stiffness between the intact and the damaged RVEs is assumed here as macroscopic damage ($D$):

4 $${D} = \frac{{K^{{{\mathrm{UND}}}} - K^{D} }}{{K^{{{\mathrm{UND}}}} }}.$$

**Fig. 4:**
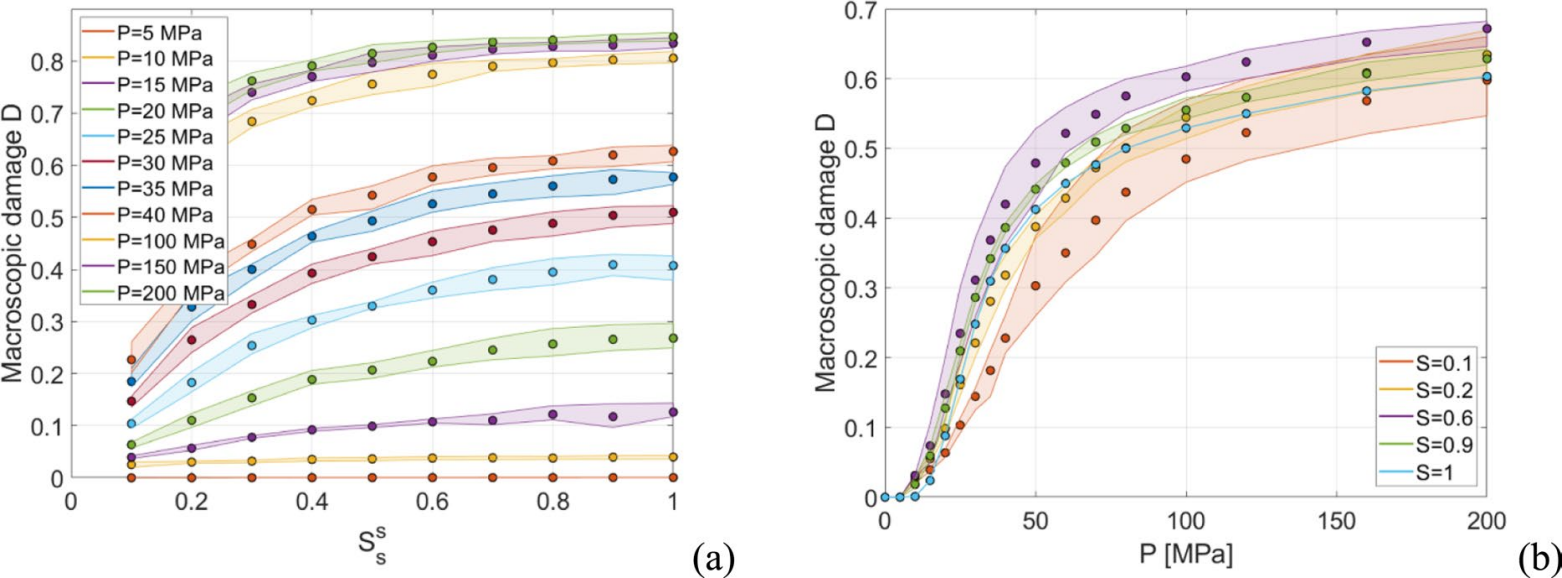
Micromechanics results used as training data for red fired clay brick. Macroscopic damage *D* results obtained by means of FE simulations **a** with constant $P$ and increasing values of $S_{s}^{s}$ and **b** with constant $S_{s}^{s}$ and monotonically increasing $P$

Three different pressure-loaded pores distributions are considered for each case (see Appendix 1 for further details), and the resulting response envelopes together with the average $D$ value (circles) are shown in Fig. 4 (for the red fired clay brick case). In particular, Fig. 4 shows the macroscopic damage $D$ results obtained by means of FE simulations with constant $P$ and increasing values of $S_{s}^{s}$ (Fig. 4a) and with constant $S_{s}^{s}$ and monotonically increasing $P$ (Fig. 4b), used in the following to train a phenomenological damage model.

**Fig. 5:**
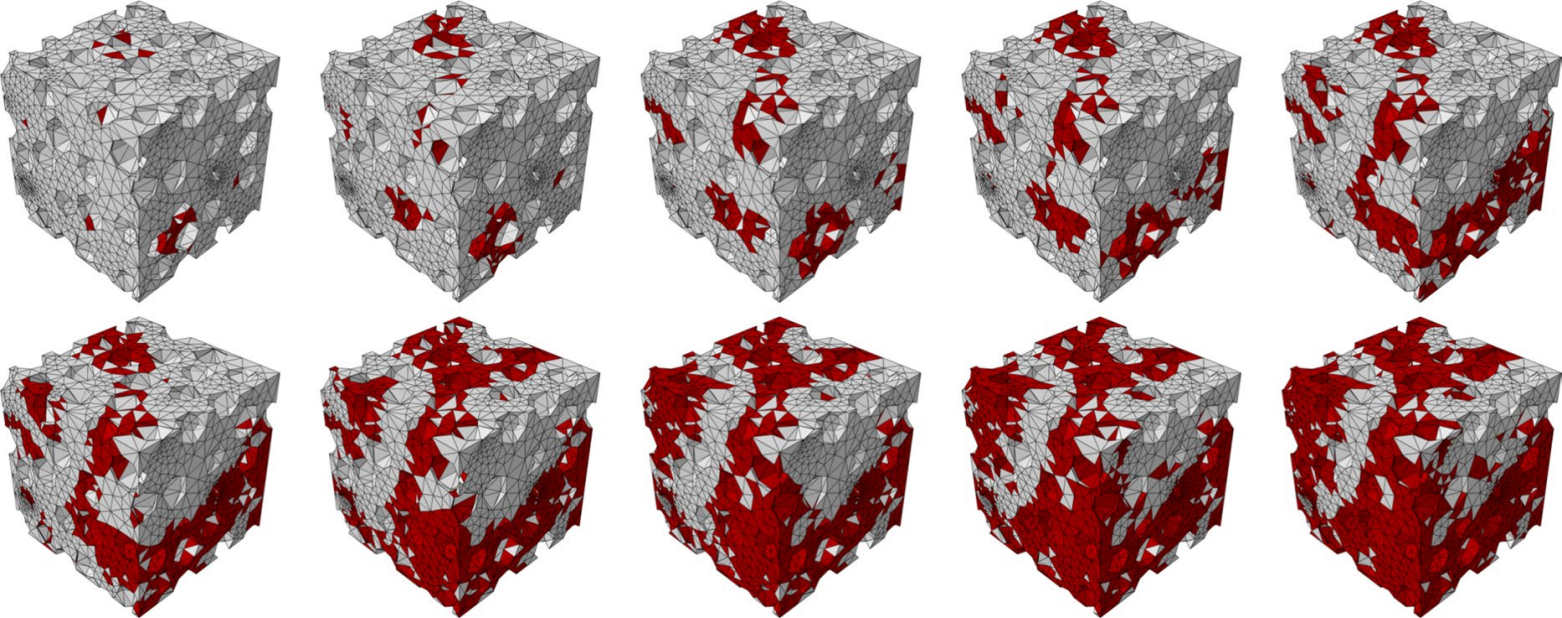
Examples of damaged RVE for the clay body of Dutch tiles case (red elements highlight a damaged condition with $0.3 \le d_{c} \le 0.9$). Various subsequent levels of ${D}$: (top line, from left to right) 0.05, 0.10, 0.15, 0.20, 0.25, (bottom line, from left to right) 0.30, 0.35, 0.40, 0.45, 0.50

Theoretically, $D$ ranges between ${D} = 0$, where the damage in the RVE is irrelevant, and the limit case ${D} = 1$, where the RVE is completely damaged, i.e., it is unable to sustain any load. However, given the confinement guaranteed in the compression test (Fig. 3c), the local damage limitation to 0.9 to guarantee numerical convergence, and the fact that local damage may not involve each portion of the RVE, the condition ${D} = 1$ is practically never reached (see, e.g., Figs. 4, 5). In particular, Fig. 5 highlights examples of RVEs with different macroscopic damage ${D}$ values, for the case of the clay body of Dutch tiles. As it can be noted, ${D}$ is substantially linked to the damage level in the RVE. In addition, small values of macroscopic damage (e.g., ${D} = 0.05$) already show some degradation in the RVE (Fig. 5), while values of ${D} > 0.3$ already show considerable damage levels on the RVE (Fig. 5).

#### Phenomenological damage model

According to the procedure described so far, given a certain pseudo-time history of $P$ and $S_{s}^{s}$ in a material point, we can evaluate the induced macroscopic damage $D$ from a micromechanical perspective. However, given that the material points to be investigated in simulations of porous construction materials subjected to salt attack might have an order of magnitude equal or greater than 10^3^, the same number of micromechanical simulations might be required. This aspect might appear an issue in terms of computational efficiency, as it might consistently limit the applications of the present approach. For this reason, we introduce a phenomenological damage model which aims at real-time estimating the macroscopic damage in a material point given a certain pseudo-time history of $P$ and $S_{s}^{s}$ (coming, e.g., from the multiphase model results), see Fig. 1. Such phenomenological damage model serves as efficient surrogate model to estimate macroscopic damage and is trained based on a FE simulation-generated dataset (see Fig. 4) connecting pseudo-time histories of $P$ and $S_{s}^{s}$ and $D$. In the following, $D$ refers to the macroscopic damage evaluated through FE simulations, while $D^{*}$ refers to the macroscopic damage estimated by means of the phenomenological damage model. Accordingly, the objective of this subsection is to provide a phenomenological damage model able to real-time predict $D^{*}$ in good agreement with $D$.

A simple phenomenological damage model is developed through the following initial value problem:

5 $$\left\{ {\begin{array}{*{20}c} {\frac{{{\mathrm{d}{D}}^{*} }}{{{\mathrm{d}}t^{*} }} = \frac{{\partial {{D}}^{*} }}{{\partial S_{s}^{s} }}\left| {\frac{{{\mathrm{d}}S_{s}^{s} }}{{{\mathrm{d}}t^{*} }}} \right| + \frac{{\partial {{D}}^{*} }}{\partial P}\max \left( {\frac{{{\mathrm{d}}P}}{{{\mathrm{d}}t^{*} }},0} \right)} \\ {D_{0}^{*} = 0 } \\ \end{array} } \right.$$

being $t^{*}$ the fictitious pseudo-time (in hours, adopted as problem independent variable), $D_{0}^{*}$ the initial value of $D^{*}$ at $t^{*} = 0$, $P$ the crystallization pressure in MPa, and $S_{s}^{s}$ the pore filling. In (5), if $\frac{{\partial D^{*} }}{{\partial S_{s}^{s} }}$ and $\frac{{\partial D^{*} }}{\partial P}$ are non-negative then $D^{*}$ increments are equal or greater than 0. On this regard, by inspecting Fig. 4, it can be noted that with the non-negativeness of $\frac{{\partial D^{*} }}{{\partial S_{s}^{s} }}$ and $\frac{{\partial D^{*} }}{\partial P}$ this requirement appears fulfilled. The goal is then to derive expressions of $\frac{{\partial D^{*} }}{{\partial S_{s}^{s} }}$ and $\frac{{\partial D^{*} }}{\partial P}$ so that the training dataset (see, e.g. Figure 4) is well-fitted. Accordingly, on the basis of the similarities of the different curves with constant $P$ and increasing values of $S_{s}^{s}$ (Fig. 4a) and with constant $S_{s}^{s}$ and monotonically increasing $P$ (Fig. 4b), the following relationships are assumed:

6 $$\frac{{\partial D^{*} }}{{\partial S_{s}^{s} }} = \alpha_{S} \left( {\frac{{\max \left( {P - P_{{{\mathrm{min}}}} , 0} \right)}}{{P_{{{\mathrm{ref}}}} }}} \right)^{{\beta_{S} }} \left( {1 - \frac{{D^{*} }}{{D_{{{\mathrm{max}}}} }}} \right)^{{\gamma_{S} }} \left( {1 - S_{s}^{s} } \right) S_{s}^{{s\eta_{s} }}$$

where $\alpha_{s}$, $\beta_{s}$, $\gamma_{s}$, and $\eta_{s}$ are parameters to be calibrated using as reference a simulation-generated dataset obtained with constant $P$ and increasing values of $S_{s}^{s}$ (Fig. 4a), $P_{{{\mathrm{min}}}}$ (minimum pore pressure to induce damage) is assumed equal to the tensile strength of the pore structure, $P_{{{\mathrm{ref}}}}$ is a reference pressure to scale the problem assumed equal to the compressive strength of the pore structure, and $D_{{{\mathrm{max}}}}$ is the maximum value of macroscopic damage observed in the simulations, here set as $D_{{{\mathrm{max}}}} = 0.9$, and:

7 $$\frac{{\partial D^{*} }}{\partial P} = \alpha_{P} \left( {\frac{{\max \left( {P - P_{{{\mathrm{min}}}} , 0} \right)}}{{P_{{{\mathrm{ref}}}} }}} \right)^{{\beta_{P} }} e^{{\frac{{\gamma_{P} }}{{D^{*} - D_{{{\mathrm{max}}}} }}}} f\left( {S_{s}^{s} } \right)$$

where $\alpha_{P}$, $\beta_{P}$, and $\gamma_{P}$ are parameters to be calibrated using as reference a simulation-generated dataset obtained with constant $S_{s}^{s}$ and monotonically increasing $P$ (Fig. 4b), and $f\left( {S_{s}^{s} } \right)$ is a function which allows to account for the tendency shown in Fig. 4b of having higher damage for $S_{s}^{s} = 0.6$ rather than $S_{s}^{s} = 1$, which is here assumed as $f\left( {S_{s}^{s} } \right) = - \,0.7S_{s}^{s3} - \,4.7S_{s}^{s2} + \,6.4S_{s}^{s}$.

**Fig. 6:**
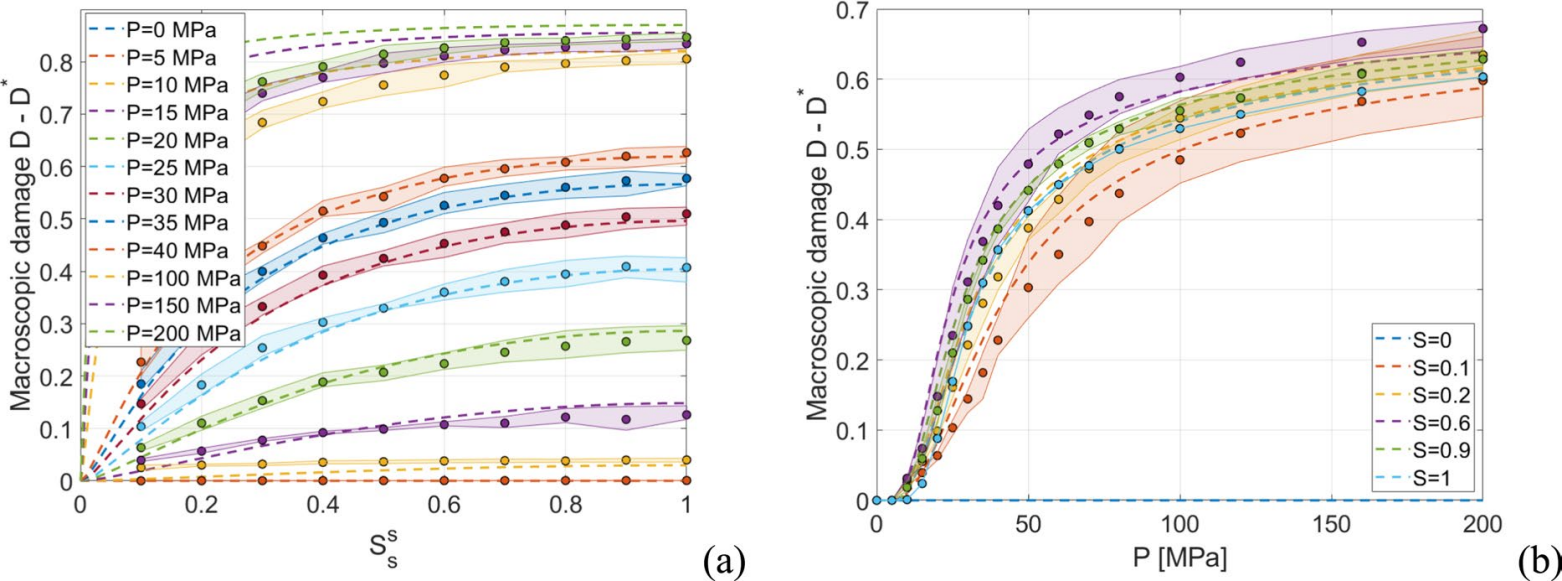
Calibration and validation of the phenomenological damage model for red fired clay brick, superimposition of training FE data (envelopes and circles) and phenomenological results (dotted lines): **a** constant $P$ with increasing values of $S_{s}^{s}$, and **b** constant $S_{s}^{s}$ and increasing $P$

Based on the training dataset generated through FE simulations (see e.g. Figure 4), the phenomenological damage model parameters are calibrated using a soft calibration strategy. In this study, phenomenological damage model parameters are calibrated for the red fired clay brick and the clay body of Dutch tiles, see Table 2. It should be highlighted that the phenomenological damage model can be used to represent any porous building material, once training data are available. As a result, accurate results are obtained as shown in Fig. 6, i.e., the $D^{*}$ curves lie within the $D$ curve envelopes.

**Table 2:** Phenomenological damage model parameters

Material	$P_{{{\mathrm{min}}}}$(MPa)	$P_{{{\mathrm{ref}}}}$(MPa)	$D_{{{\mathrm{max}}}}$	$\alpha_{s}$	$\beta_{s}$	$\gamma_{s}$	$\eta_{s}$	$\alpha_{P}$	$\beta_{P}$	$\gamma_{P}$
Red fired clay brick	6.8	20.3	0.9	3.1	1.9	2.4	0.3	0.55	0.8	2.6
Clay body of Dutch tiles	6	21	0.9	0.1	2.4	2.1	0.05	0.006	0.9	1.6

**Fig. 7:**
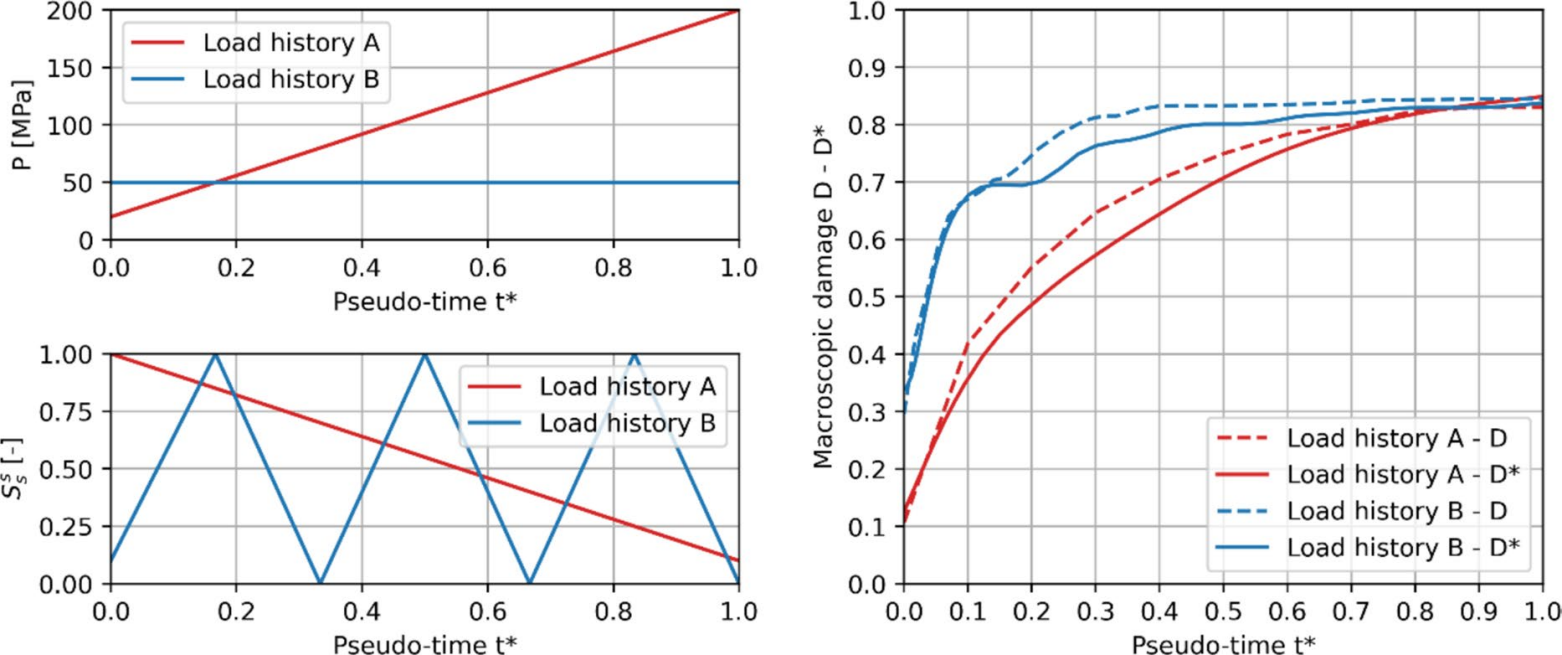
A posteriori validation of the phenomenological damage model. Examples of two pseudo-time load histories (left) and corresponding macroscopic damage (right) for clay body of Dutch tiles. FE-evaluated macroscopic damage $D$ is shown by means of solid lines, while the estimated macroscopic damage $D^{*}$ is shown by means of dotted lines

The capability of the phenomenological damage model to predict macroscopic damage due to pseudo-time histories of $P$ and $S_{s}^{s}$ not considered in the training dataset is further investigated. In particular, several scenarios with varying both $P$ and $S_{s}^{s}$ are considered, together with cases with cyclic variations. For the sake of conciseness, only two cases are shown in Fig. 7, showing an accurate prediction of the phenomenological damage model even for cases not used in the training dataset. Accordingly, the phenomenological damage model allows real-time accurate predictions of macroscopic damage in a material point given any pseudo-time history of $P$ and $S_{s}^{s}$ and is used in the following to provide mechanical damage information along with simulations of salt transport and crystallization in porous building materials.

It should be pointed out that the proposed phenomenological damage model represents the simplest real-time predictor developable with the available dataset. It appears to be robust, even enclosing physical aspects. Anyway, more advanced tools based on simulation-driven machine learning algorithms could be developed following an akin framework, also considering larger and richer datasets (even hybrid numerical-experimental datasets). Finally, it has also to be pointed out that the present study presents of a one-way field transfer from the multiphase model to the phenomenological model to compute the damage. In any case, a two-way approach (see, e.g., [32]) through which the hygric properties of the porous material are updated step-by-step based on the computed damage could be straightforwardly implemented in the same framework, once the laws that relates damage with hygric properties are made available.

## Results and discussion

### Proof of concept

**Fig. 8:**
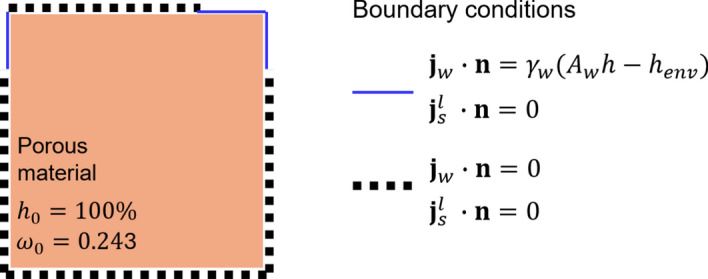
Proof-of-concept benchmark. Boundary conditions for a drying simulation

**Fig. 9:**
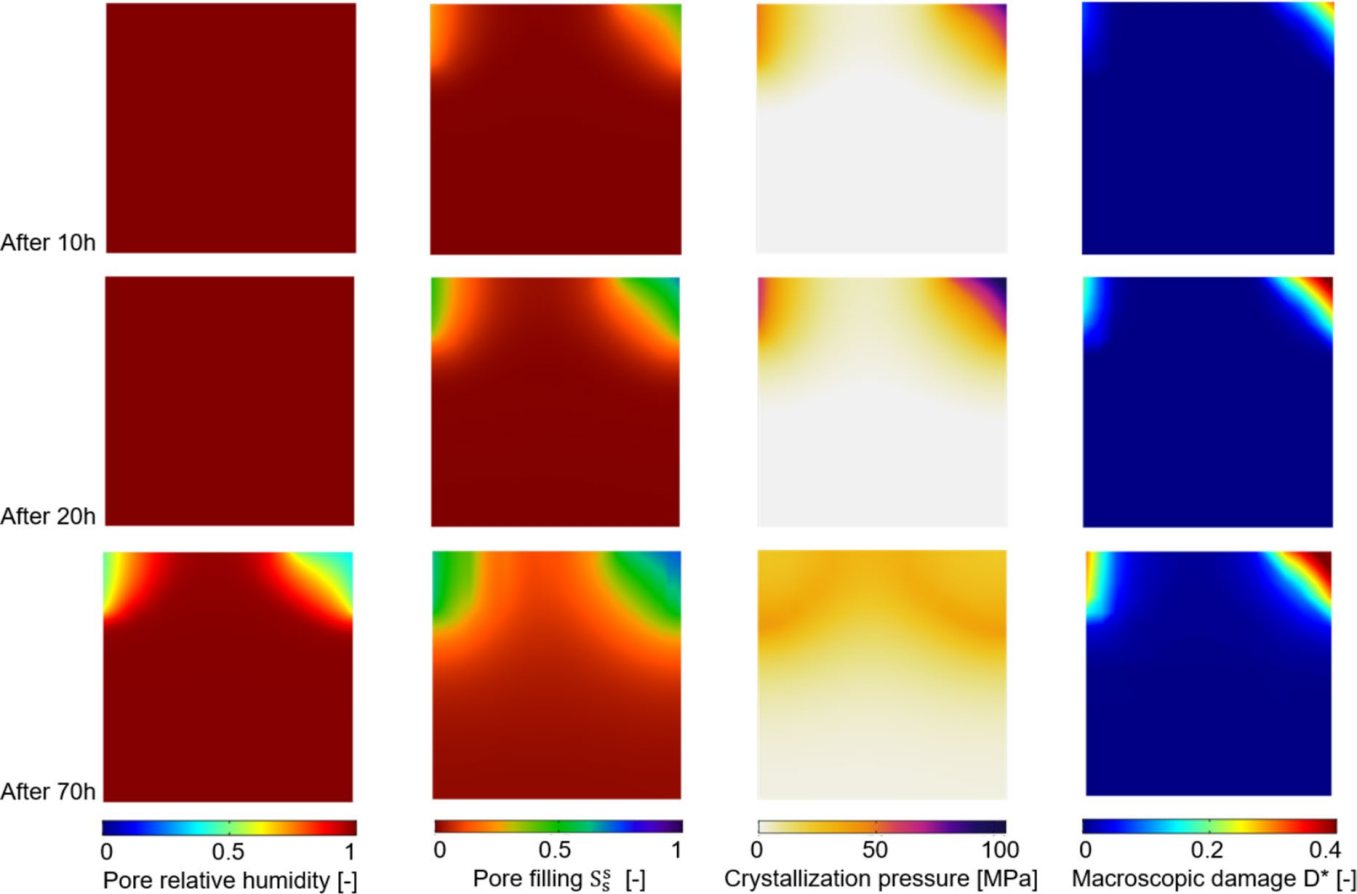
Proof-of-concept benchmark results. Evolution of pore relative humidity (first column), pore filling $S_{s}^{s}$ (second column) and crystallization pressure $P$ (third column) at subsequent steps of the drying simulation, along with the evolution of the macroscopic damage $D^{*}$ (last column)

To show the potential and the effectiveness of the adopted numerical approach, the simple benchmark shown in Fig. 8 is used as proof of concept for the proposed multiscale modelling strategy to simulate salt crystallization-induced damage in porous building materials. A vertical cross-section of a 8 $\times$ 8 mm squared clay body, discretized by means of quadrangular quadratic FEs with an average size of 0.2 mm, of Dutch tiles specimen fully saturated with a $\omega = 0.243$ NaCl solution is considered, allowing the water liquid flux only in two parts of the boundary as shown in Fig. 8 considering an environmental relative humidity of 25%. Figure 9 shows the results of the drying simulation (multiphase results) together with the mechanical damage. In particular, Fig. 9 shows the evolution of pore relative humidity, pore filling $S_{s}^{s}$ and crystallization pressure $P$ at subsequent steps of the drying simulation, along with the evolution of the macroscopic damage $D^{*}$ computed real-time by the phenomenological damage model, according to the workflow shown in Fig. 1. As it can be noted, macroscopic damage $D^{*}$ monotonically evolves specially in the specimen top corners reaching considerable levels of damage (e.g., $D^{*} = 0.4$, see for reference Fig. 5) where higher levels of $P$ and $S_{s}^{s}$ were obtained by means of the multiphase simulation. Accordingly, the proposed approach appears appealing to effectively and efficiently simulate salt crystallization-induced damage in porous building materials.

### Experimental campaign used as reference

In the following, we use as reference the experimental campaign on salt weathering in antique Dutch ceramic tiles discussed in [36]. In particular, prismatic 13 cm × 13 cm samples of Dutch tiles with an average height of 8 mm, including a glaze layer of 0.3 mm were considered. Sub-samples were cut from these tiles considering either two-layered samples or clay body only samples. Salt weathering cyclic tests inspired by [20] and performed through repeated accelerated wetting–drying experiments utilizing sodium chloride (NaCl), which crystallizes in its anhydrous form (halite), are here considered. A salt solution concentration equivalent to 90% of the saturated concentration at room temperature (i.e., a 5.5 molal solution of sodium chloride) is adopted. Prior to the weathering experiments, microstructural, hygric and mechanical characterizations of the samples were performed, and the results are detailed in [36]. The hygric characterization includes the determination of the drying kinetics of two-layered and clay body samples saturated with pure water (collected in Appendix 2), as well as the drying kinetics of two-layered samples saturated with NaCl solution, as discussed in the following subsection. For each test series, five samples were used, and drying took place at 25% RH and 21 °C.

**Fig. 10:**
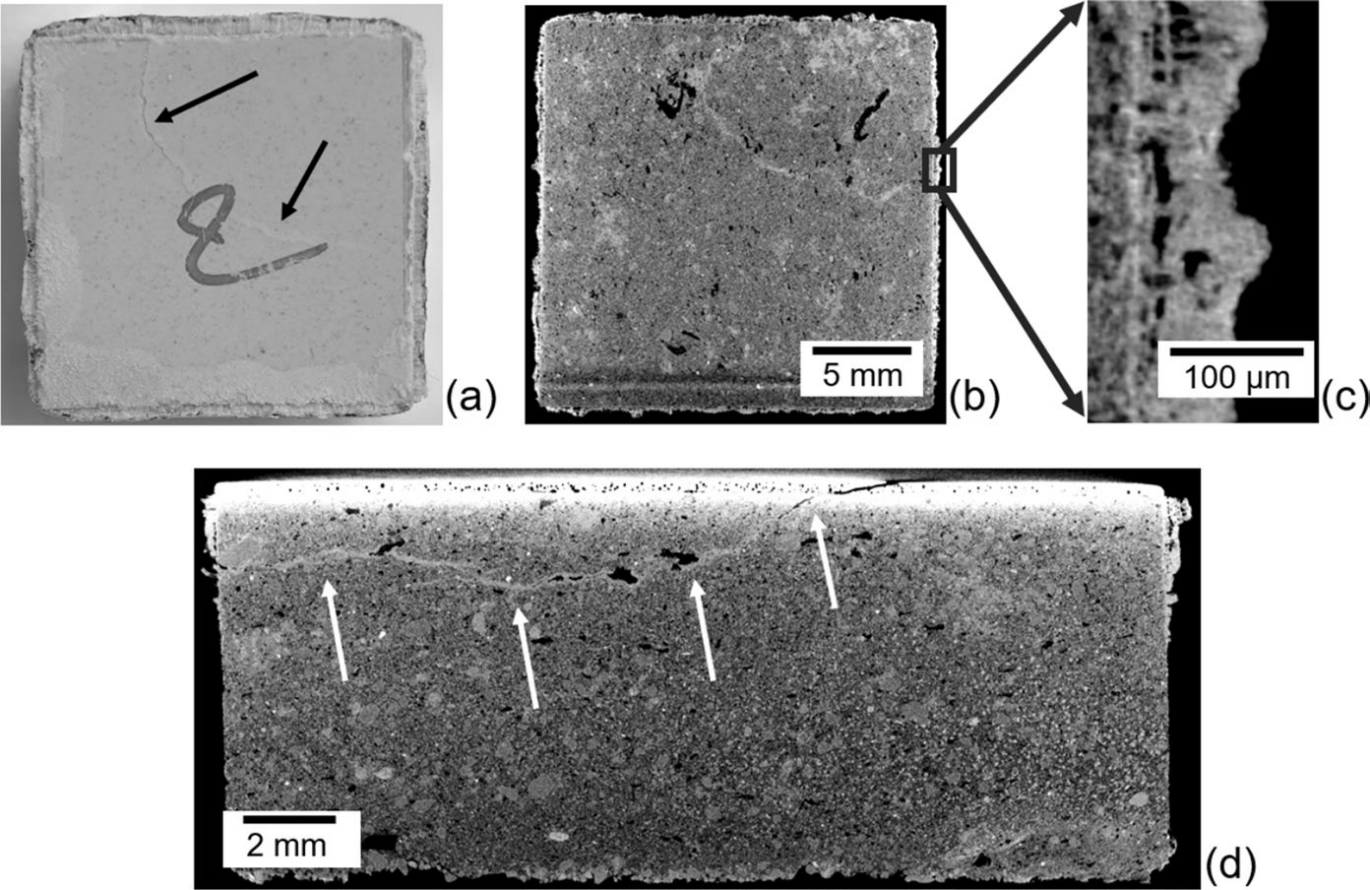
X-ray micro-computed tomography results at the end of the drying experiments on two-layered samples saturated with NaCl, adapted from [36]. **a** Initially intact tile sample damaged by NaCl precipitation (crack in the glaze highlighted by black arrows), **b** horizontal cross-section of the clay body, **c** salt crust at the border of the sample, **d** vertical cross-section of the sample showing damage induced by NaCl precipitation (crack in the body highlighted by white arrows)

Experimentally, the intact glaze resulted impermeable (see [36]), and the drying took place between the sample and the lateral aluminium sealing (Fig. 10), so mimicking the actual drying process of tiles installed on walls. In particular, two-layered samples saturated with NaCl showed remarkably slow drying behaviours due to the formation of NaCl crust on the surface of drying [36], and the drying only occurred through the crust (Fig. 10). It should be highlighted that two of the two-layered samples contaminated with NaCl out of five experienced macroscopic damage during the drying process. An example is showed through X-ray micro-computed tomography (carried out at the end of the weathering process) in Fig. 10 (right), where a crack crosses glaze and clay body following several large pores located in the clay body near to the glaze, suggesting that the damage was significantly influenced by pre-existing defects [36], i.e., defects in the glaze and large pores coarsely aligned in the clay body.

### Comparison with experimental outcomes

**Fig. 11:**
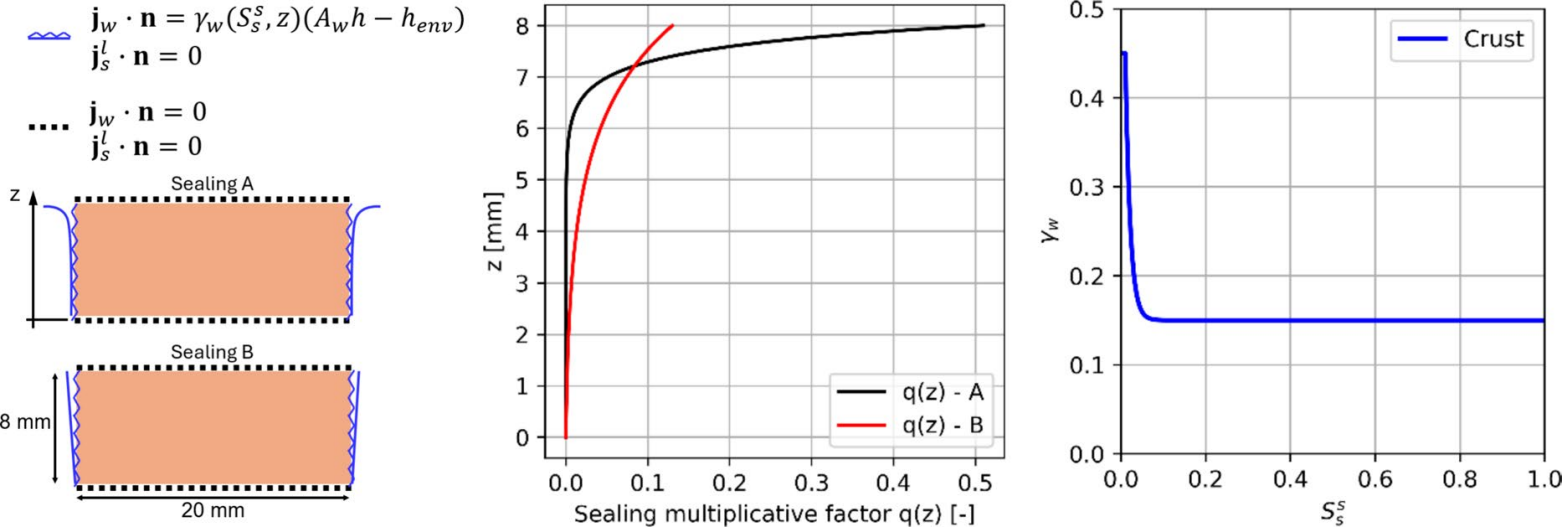
Boundary conditions for the two-layered sample contaminated with NaCl solution (left). Variation of $\gamma_{w}$ along the height of the sample to replicate the imperfect sealing observed experimentally (middle). Variation of $\gamma_{w}$ as a function of $S_{s}^{s}$ to numerically represent the NaCl crust growth (right)

Drying experiments on Dutch tiles samples are simulated by means of the proposed multiscale modelling strategy. Giving the substantial impermeability of the glaze, it has been idealized as a zero-thickness no-flux surface. In particular, a thin vertical slice representative of an internal condition of the sample is modelled. Multiphase model parameters have been set according to the experimental outcomes in [36], considering also the drying kinetics of clay body and two-layered samples, see Appendix 2. It should be highlighted that, according to [36], the lateral aluminium sealing resulted imperfect, as the drying process took place between the aluminium tape and the sample, leading to a nontrivial modelling of the lateral boundary conditions in the multiphase model. For this reason, two hypotheses of imperfect sealing are made (Sealing A and Sealing B, see Fig. 11 and Appendix 2 for further details), supposing the convective humidity coefficient $\gamma_{w}$ to vary along with the specimen height, as shown in Fig. 11. Also, the formation of NaCl crust in the drying surface between the sealing and the sample, which leads to remarkably slow drying behaviours, makes nontrivial the idealization of the lateral boundary conditions. The approach proposed in [52] to account for the presence of salt crust on the convective humidity coefficient $\gamma_{w}$ has been herein considered. In particular, $\gamma_{w}$ has been adopted to decrease while increasing $S_{s}^{s}$, according to [52], as shown in Fig. 11. As it can be noted, $\gamma_{w}$ drops rapidly as soon as $S_{s}^{s}$ increases, reaching a residual value equal to 1/3 of its initial value at around $S_{s}^{s}$ = 0.1. This aims at reproducing the effects of salt crust formation between the lateral sealing and the sample. Accordingly, the convective humidity coefficient $\gamma_{w}$ in Eq. (1) is replaced by $\gamma_{w} = q\left( z \right) \gamma_{w} \left( {S_{s}^{s} } \right)$, i.e., the convective humidity coefficient in the lateral surfaces is assumed to vary along with the height of the sample and to depend upon the pore filling in the portions close to the lateral surfaces.

**Fig. 12:**
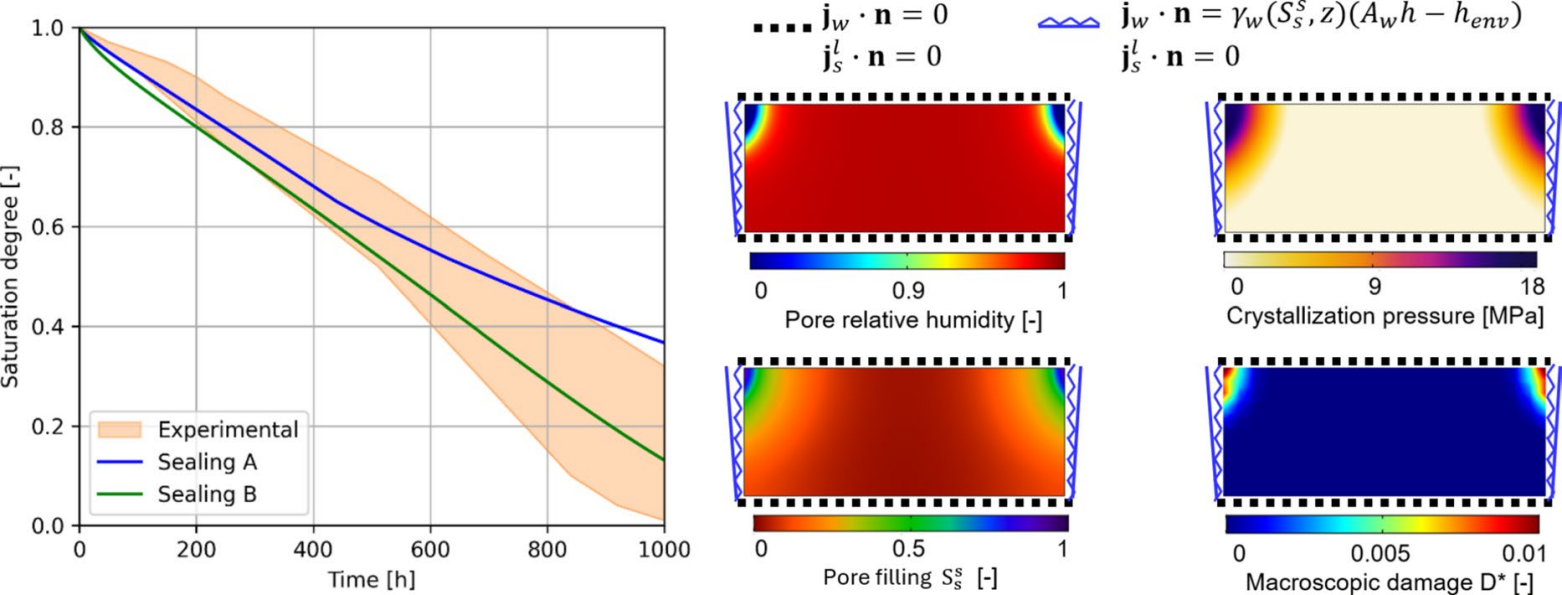
Drying behavior of the two-layered sample contaminated with NaCl solution. Comparison of experimental and numerical drying curves (left). Contour plots (right) of pore relative humidity $h$, crystallization pressure $P$, pore filling $S_{s}^{s}$, and macroscopic damage $D^{*}$ at the end of the drying simulation. Simulation time on a computer equipped with an Intel Core i7-10510U and 16 GB RAM: 41 min 20 s (Sealing A), 07 min 18 s (Sealing B)

To estimate macroscopic damage along with the simulations, the phenomenological damage model set according to clay body (Dutch tile) in Table 2 is adopted. Drying simulations are then run and the macroscopic damage $D^{*}$ is tracked along with the multiphase analysis progress. The resulting drying curves are shown and compared with the experimental envelope in Fig. 12 for both Sealing A and Sealing B cases without considering any defect. As it can be noted, both hypotheses on imperfect sealing appear plausible being mostly included within the experimental envelope, with Sealing A drying slightly slower than Sealing B, especially in the second half of the drying simulation (Fig. 12). The contour plots of pore relative humidity $h$, crystallization pressure $P$, pore filling $S_{s}^{s}$, and macroscopic damage $D^{*}$ at the end of the drying simulation are also shown in Fig. 12. As expected, the top corners resulted in the most significant parts in terms of drying (lowest value of $h$), crystallization pressure (highest value of $P$) and pore filling (highest value of $S_{s}^{s}$). In particular, a significant solid salt accumulation is recorded, especially in the zones close to the top corners. Although the macroscopic damage $D^{*}$ resulted different from zero around the top corners, significant damage levels were not foreseen being $D^{*}$ always smaller than 0.01. Such outcomes appear in agreement with the experiments in the hypothesis of absence of defects, as the majority of samples did not show observable damage at the end of the drying phase.

**Fig. 13:**
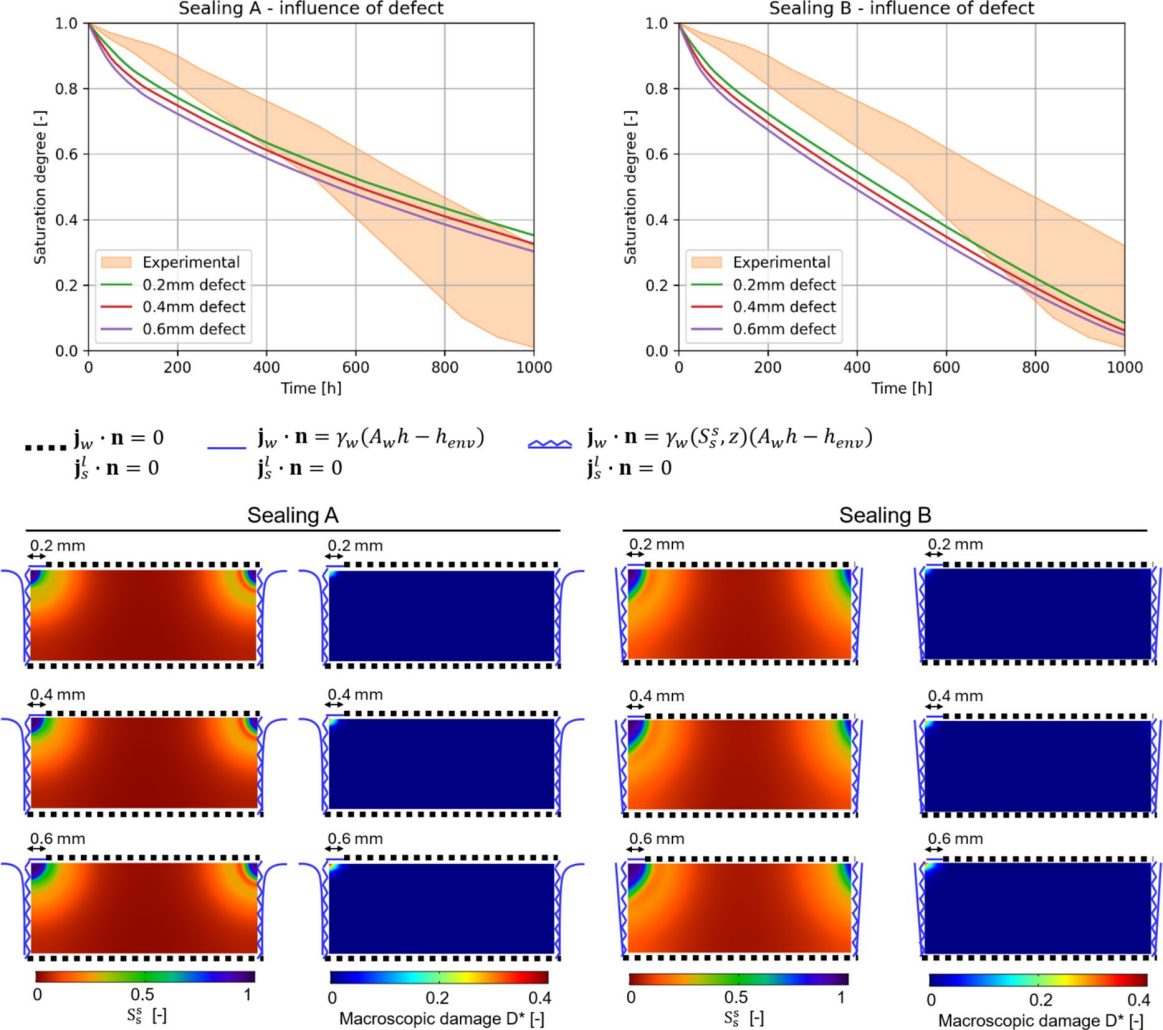
Drying behavior of the two-layered sample contaminated with NaCl solution considering defects

To further investigate the defect-induced damage triggering in the samples (as noted experimentally in [36]), the same drying simulations are then run by parametrically considering defects on one top corner (particularly, on the top surface of the left one, see Fig. 13). Defects of 0.2 mm, 0.4 mm, and 0.6 mm are considered by specifying free liquid flux on the defect surface, mimicking a crack in (or a detached portion of) the glaze. Both Sealing A and Sealing B are considered.

**Fig. 14:**
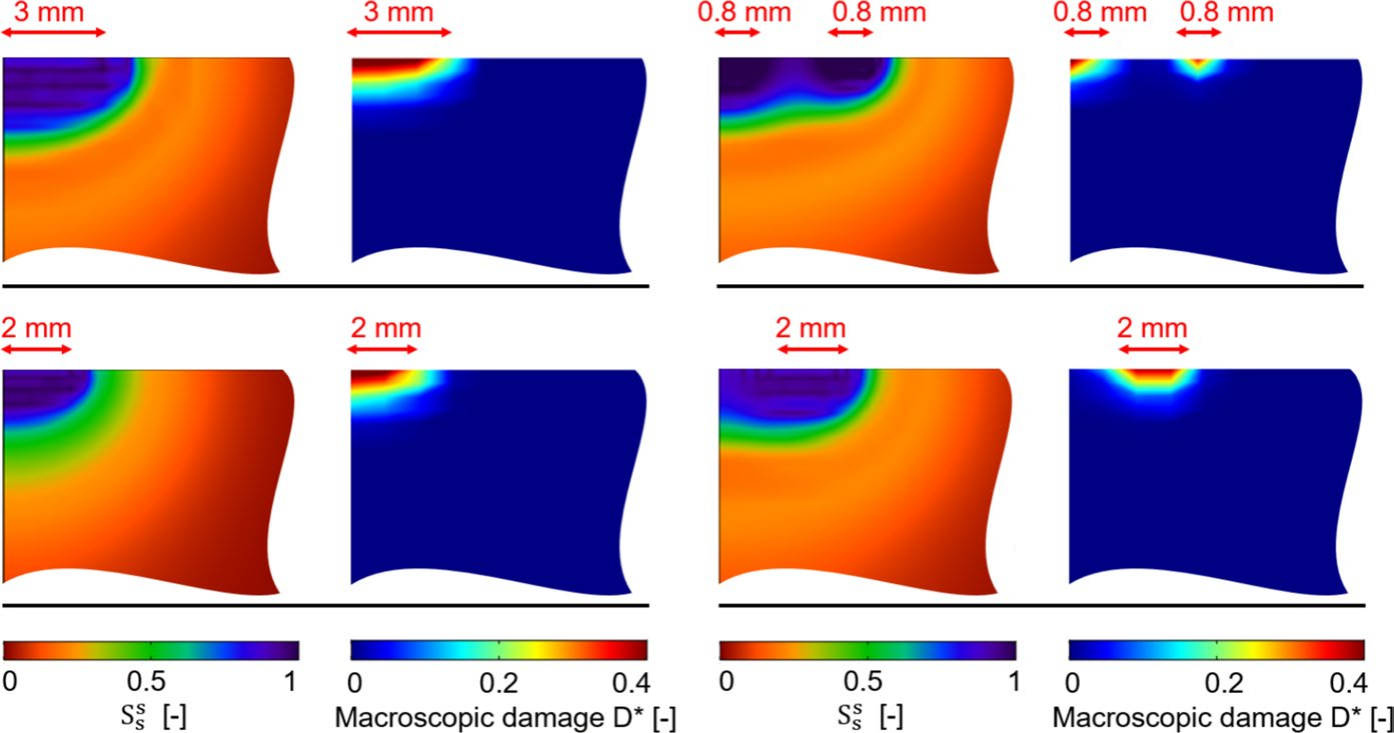
Scenarios of defect-induced damage in drying two-layered samples with NaCl solution. Magnified portion of the top left corner, where irregular right and bottom edges indicate cut parts

As can be observed from the drying curves in Fig. 13, the defect effects on the drying curves appear perceptible, although globally limited. By inspecting the pore filling contour plots in Fig. 13, it can be noted that solid salt accumulation slightly increases in the top left corner while increasing the defect size, for both Sealing A and Sealing B. It appears worth to note that the macroscopic damage becomes significant as soon as a defect is introduced in the sample and reaches high values of $D^{*}$ (up to 0.4, see Fig. 5) in the top left corner for 0.4 mm and 0.6 mm defect sizes. These outcomes highlight how damage can be triggered by the presence of defects in the glaze. Additional defects scenarios which further point out this aspect are shown in Fig. 14, where larger or multiple defects are considered. As it can be observed, defects might even induce damage beneath no flux surfaces (such as glaze or perfect sealings), so significantly modifying the sample set-up and posing the basis for damage progression and glaze detachment.

**Fig. 15:**
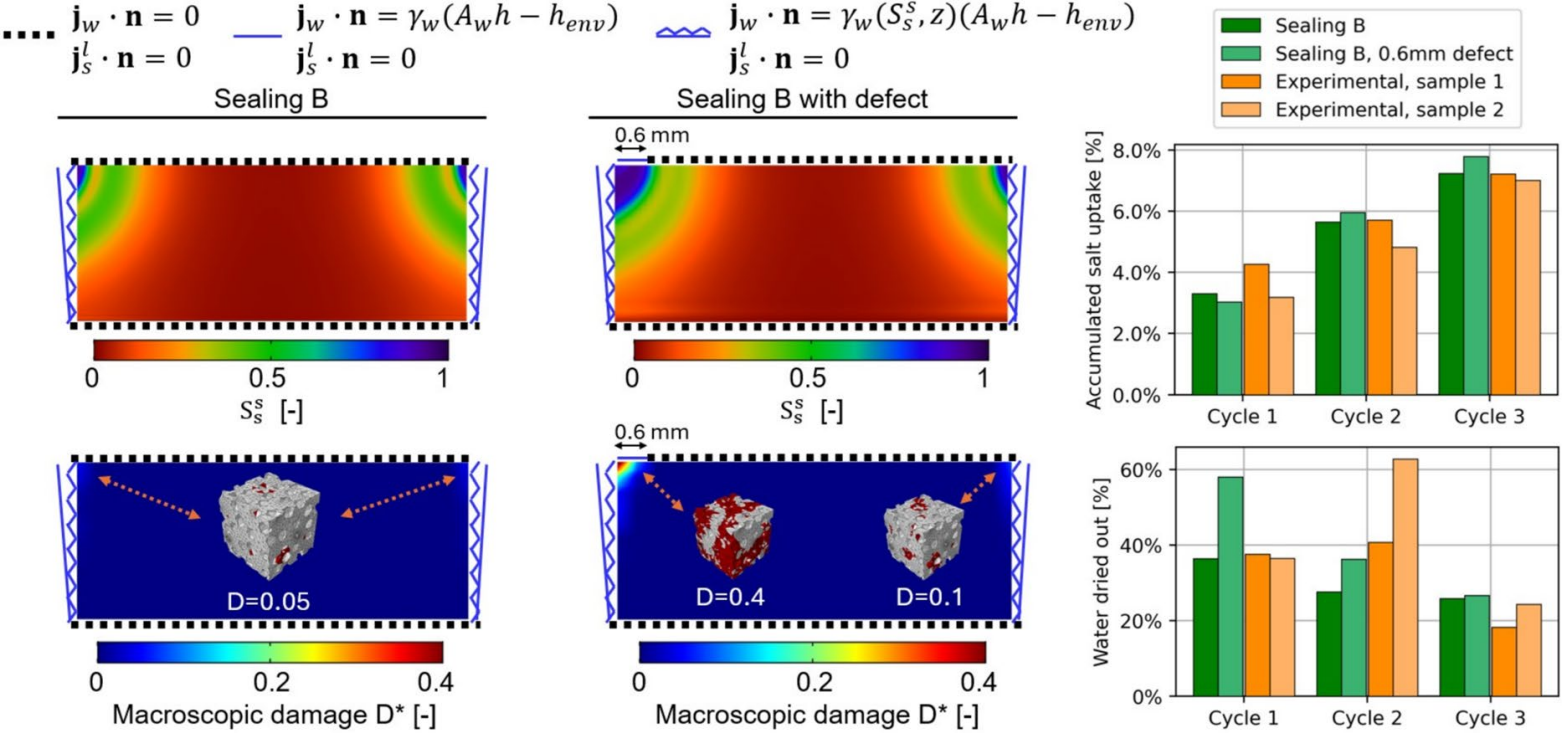
Two-layered sample contaminated with NaCl solution subjected to three weathering cycles. Contour plots of pore filling $S_{s}^{s}$ and macroscopic damage $D^{*}$ at the end of weathering for the case without (left) and with (middle) defect. Experimental–numerical comparison in terms of accumulated salt uptake and amount of water evaporated at the end of each drying phase

Finally, the two-layered samples contaminated with NaCl solution and subjected to three weathering cycles (7-day wetting–drying cycles with drying at 25% RH [36]) are reproduced numerically and compared with the experimental results, see Fig. 15. In particular, the experimental–numerical comparison is carried out in terms of accumulated salt uptake and water evaporated at the end of each drying phase. Overall, a good agreement between numerical and experimental accumulated salt uptake can be observed. The numerically derived amount of water evaporated during the first weathering cycle of the case with defect appears higher than the case without defect, as well as experimental values, as the defect allows a higher exchange of water with the environment. This trend is then smoothed in the following cycles as the accumulation of crystallized salt in the upper-left zone of the simulated sample reduces the flux of water. In addition, it should be highlighted that the experimental outcomes present an outlier in terms of water evaporated during the second cycle from sample 2. Plausibly, this higher value might have been caused by defects in the sealing that was applied and then removed at each cycle of the experimental test. This evidence might highlight the significant sensitivity to boundary conditions of both numerical and experimental results. Although macroscopic damage does not increase significantly along with the cycles (independently by the presence of defects) as also experienced experimentally [36], the solid salt accumulation progresses, and the pore filling contour plots show higher salt crystallization with respect to one drying phase only. All things considered, the proposed multiscale modelling strategy to simulate salt crystallization-induced damage in porous building materials appeared effective and able to foresee even damage triggered by defects. Furthermore, the use of the phenomenological damage model to estimate macroscopic damage appeared particularly efficient.

## Conclusions

In this paper, a multiscale modelling strategy has been proposed to efficiently simulate salt crystallization-induced damage in porous materials. Salt crystallization pressure exerted on pore walls has been explicitly modelled on a RVE of the porous medium, accounting for the confinement provided by the surrounding material. The well-known law of partial pressures has not been adopted here, as the micromechanical model allowed for a direct definition of the number of pores to be loaded. A macroscopic damage measurement of the whole RVE has been obtained for several combinations of crystallization pressure and pore filling time histories.

Moisture and salt transport, and salt crystallization in porous media has been simulated by means of a state-of-the-art multiphase model, from which crystallization pressure and pore filling pseudo-time histories could be extracted through simple post-processing in any point of the macroscopic domain. The efficient coupling of the multiphase model and micromechanical damage has been achieved by originally formulating a phenomenological damage model, trained on a dataset generated through micromechanics-based simulations on RVEs.

The effectiveness of the proposed numerical strategy has been shown firstly through a proof-of-concept example, and then via the comparison with an experimental campaign on salt-aged traditional Dutch tiles. The proposed multiscale approach could indeed predict the occurrence of damage in two-layered samples based on the multiphase model outcomes, even investigating the damaged triggered by defects in the samples, as well as the effects of weathering cycles.

Future developments could regard the specialization of the phenomenological damage model with more advanced simulation-driven machine learning tools, guaranteeing real-time macroscopic damage predictions based on databases which can be enriched to account for several conditions, different RVE outcomes, as well as hybrid numerical-experimental datasets.

## Figures and Tables

**Fig. 16:**
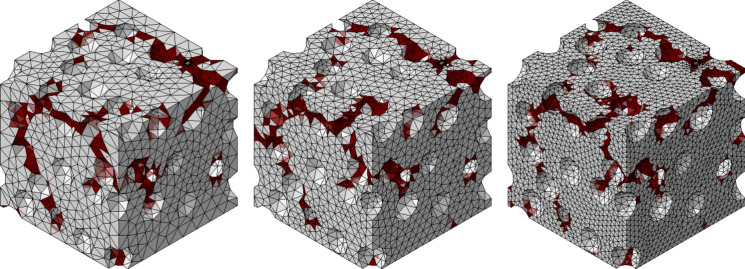
Influence of mesh size on the macroscopic damage. Average mesh size equal to $0.7$ μm (left, with 23,691 FEs), $0.35$ μm (middle, with 45,476FEs), and $0.18$ μm (right, with 82,399FEs)

**Fig. 17:**
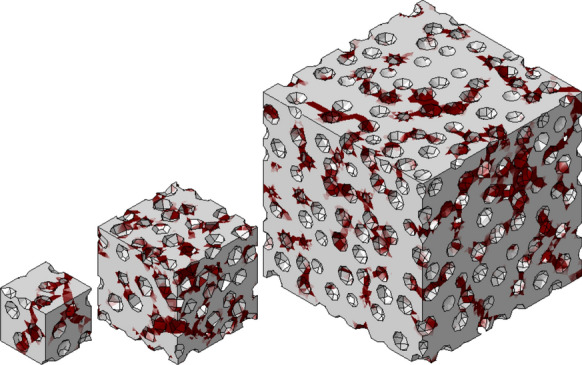
Influence of RVE size. RVE sizes equal to $4$ μm (left), $7$ μm (middle), and $14$ μm (right). Damage due to a crystallization pressure of $35\,{\mathrm{MPa}}$

**Fig. 18:**
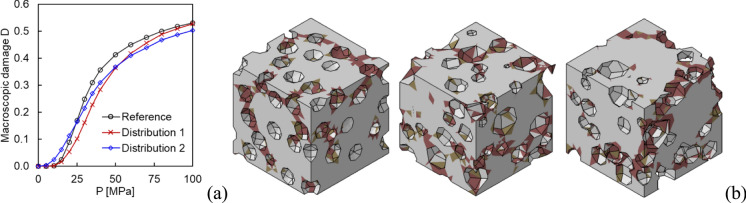
Influence of pore distribution geometry, with $S_{s}^{s} = 1$ and multiple values of $P$. **a** Evolution of macroscopic damage along with the maximum value of crystallization pressure $P$. **b** Examples of damaged RVEs due to a crystallization pressure of $35\,{\mathrm{MPa}}$ for Reference (left), Distribution 1 (middle), and Distribution 2 (right) cases

**Fig. 19:**
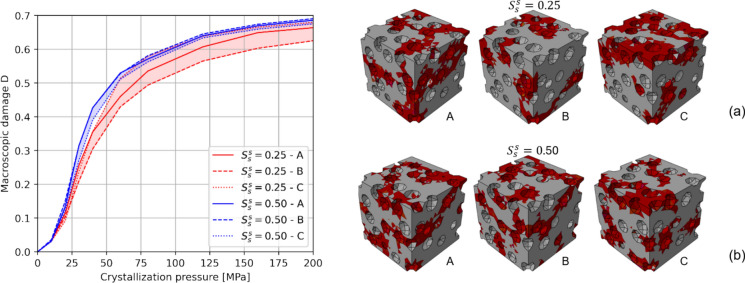
Influence of distribution of pressure-loaded pores with multiple values of $P$. Evolution of macroscopic damage along with the maximum value of crystallization pressure $P$ (left). Examples of damaged RVEs due to a crystallization pressure of $60\,{\mathrm{MPa}}$ for three different distributions of pressure-loaded pores (right) by considering a pore filling equal to **a** $S_{s}^{s} = 0.25$ and **b** $S_{s}^{s} = 0.5$

**Fig. 20:**
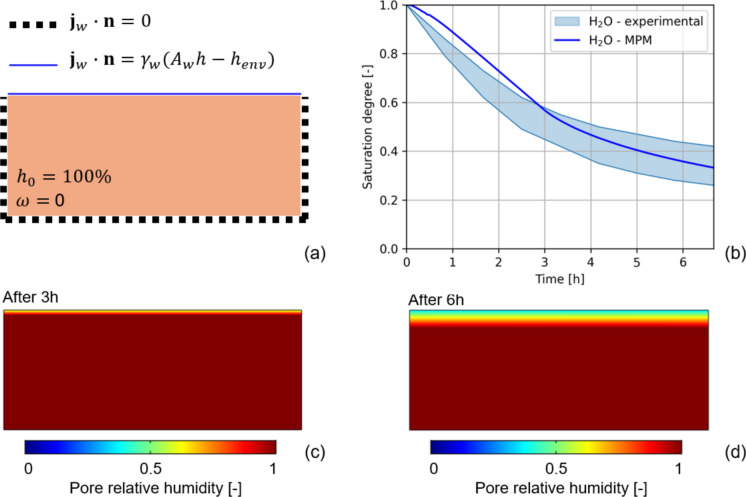
Drying simulation of the clay body saturated with pure water: **a** boundary conditions, **b** drying curves comparison between numerical and experimental outcomes, **c** pore relative humidity contour plot after 3 h of drying, **d** pore relative humidity contour plot after 6 h of drying

**Fig. 21:**
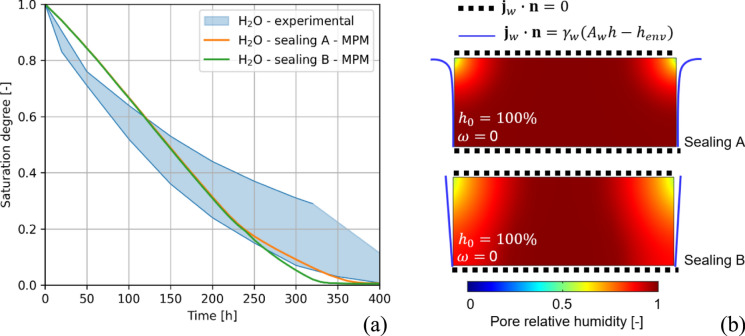
Drying simulation of the two-layered Dutch tile samples saturated with pure water. **a** Drying curves comparison between numerical and experimental outcomes. **b** Pore relative humidity contour plot after 300 h of drying for Sealing A and Sealing B hypotheses

## Appendix 1

In this appendix, parametric studies on the RVE investigating the influence of mesh size, RVE size, pore distribution geometry, and distribution of pressure-loaded pores are presented and discussed, by adopting as material the fired clay red brick in Table 1.

Firstly, the influence of mesh size is investigated with respect to the macroscopic damage. $7$ μm-RVEs discretized with average mesh sizes equal to $0.7$ μm, $0.35$ μm, and $0.18$ μm are considered and compared (Fig. 16), considering a single cycle with a crystallization pressure equal to $P = 35\,{\mathrm{MPa}}$ and $S_{s}^{s} = 1$. As it can be noted, the damage pattern on the RVE appeared sensibly akin, and the macroscopic damage resulted very similar, i.e., $D = 0.31$ for the case with $0.7$ μm, $D = 0.33$ for the case with $0.35$ μm, and $D = 0.32$ for the case with $0.18$ μm. As a result, the adopted feature to describe macroscopic damage appears almost insensible to the mesh size. Accordingly, the average mesh size equal to $0.7$ μm (i.e., equal to the mean pore radius $r_{p}$) is adopted to guarantee accuracy and computational efficiency.

Secondly, the influence of the RVE size is investigated with respect to the macroscopic damage and the variability of stiffness in the different directions (Fig. 17). In particular, three RVE sizes are considered, namely $4$ μm, $7$ μm, and $14$ μm by using the same target geometrical properties for the pores and pore distribution and average mesh size equal to $0.7$ μm. Concerning the undamaged RVEs, an average relative error of 5.8% on the stiffness is recorded for the $4$ μm-RVE with respect to the $14$ μm-RVE, while an average relative error of 1.2% on the stiffness is recorded for the $7$ μm-RVE with respect to the $14$ μm-RVE. This already points out that the $7$ μm-RVE appears a good compromise between accuracy and computational efficiency. This is further confirmed by considering a single cycle with a crystallization pressure equal to $P = 35\,{\mathrm{MPa}}$ and $S_{s}^{s} = 1$ (Fig. 17). Indeed, the average macroscopic damage measured on the $4$ μm-RVE resulted $D = 0.23$ (being the macroscopic damage in the 3 directions equal to $0.30,\,0.19,$ and $0.22$), on the $7$ μm-RVE resulted $D = 0.27$ (being the macroscopic damage in the 3 directions equal to $0.24,\,0.31,$ and $0.27$), and on the $14$ μm-RVE resulted $D = 0.26$ (being the macroscopic damage in the 3 directions equal to $0.27,\,0.25$, and $0.25$). Accordingly, $7$ μm-RVE (i.e., an RVE with edge sizes 10 times larger than $r_{p}$) reveals to be a good compromise between accuracy (e.g., smaller variations of macroscopic damage in the 3 directions and higher accuracy with respect to the $4$ μm-RVE) and computational efficiency. As a way of example, the $7$ μm-RVE simulation with a single cycle and crystallization pressure equal to $P = 35\,{\mathrm{MPa}}$ and $S_{s}^{s} = 1$ required 1 h and 21 min on a laptop equipped with a 11th Gen Intel(R) Core(TM) i9-11900H @ 2.50 GHz processor with 64 GB RAM.

Thirdly, the influence of the pore distribution geometry is investigated with respect to the macroscopic damage (Fig. 18). The case with non-superimposing spherical pores with identical radius (equal to $r_{p}$), denoted as “Reference” case, is compared with a normal distribution of non-superimposing spherical pores with standard deviation in the pore radius of $\sigma = 0.25$ μm, denoted as “Distribution 1”, and with a normal distribution of superimposing spherical pores with standard deviation in the pore radius of $\sigma = 0.15$ μm, denoted as “Distribution 2”, see Fig. 18. As it can be noted, although the substantial differences in the pore distribution geometry, the evolution of macroscopic damage along with the maximum value of crystallization pressure appears rather similar (Fig. 18a) for the three distributions considered (see examples of damaged RVEs in Fig. 18b). This outcome suggests that the reference distribution with non-superimposing identical spherical pores appears to be a simple and reasonable choice to be used within the proposed numerical approach.

Finally, the influence of pressure-loaded pores distribution is investigated with respect to the macroscopic damage (Fig. ^19^). The same RVE is loaded by considering three different random distributions of pressure-loaded pores by considering a pore filling equal to $S_{s}^{s} = 0.25$ (Fig. 19a) and $S_{s}^{s} = 0.5$ (Fig. 19b). Various values of crystallization pressure are applied, and the results are compared in Fig. 19. As it can be observed, although the RVEs considered show different damage patterns as different schemes of pressure-loaded pores are considered, the evolution of macroscopic damage along with the maximum value of crystallization pressure results rather similar in all cases. This aspect suggests that the random distribution of pressure-loaded pores does not have a significant impact on macroscopic damage.

## Appendix 2

In this appendix, the multiphase model settings calibration for Dutch tile clay body [36] are presented. Based on experimental tests, the active capillary porosity $\phi_{0} = 26.6\%$ and the water adsorption coefficient $A_{{{\mathrm{cap}}}} = 0.081\frac{kg}{{m^{2} s^{0,5} }}$ have been set in the multiphase model. The drying behaviour of Dutch tile clay body samples is reproduced numerically, see Fig. 20, by adopting a constant convective humidity coefficient $\gamma_{w} = 0.45$. As it can be noted, this setting guarantees good estimates of the drying behaviour of the clay body saturated with pure water.

Then, reference has been made to the two-layered Dutch tile samples saturated with pure water (Fig. 21). Given the substantial impermeability of the glaze (top surface), the drying process takes place between the sealing in the lateral surfaces and the sample [36]. For this reason, two hypotheses of imperfect sealing, i.e., Sealing A and Sealing B, are made and are tuned so that to reproduce the drying behaviour of two-layered Dutch tile samples saturated with pure water. In particular, Sealing A and Sealing B hypotheses involve a height-varying convective humidity coefficient $\gamma_{w}$, as shown in Fig. 11. Figure 21 shows that both Sealing A and Sealing B hypotheses result representative of the drying behaviour with pure water and are used in more complex simulations concerning salt crystallization and salt crust formation (see, e.g., Fig. 12). In particular, the model parameters for NaCl solution are adopted identically to [31], where the interested reader is referred to. Furthermore, the salt diffusion coefficient is assumed to depend on the salt solution saturation degree $S_{ws}^{l}$ which is assumed as function of the pore relative humidity $h$ through the sorption/desorption curve $S_{ws}^{l} \left( h \right)$, as in [31], which has been here derived from experiments [36].
